# Geospatial Analysis of Food Deserts and Their Impact on Health Outcomes in Children with Cystic Fibrosis

**DOI:** 10.3390/nu13113996

**Published:** 2021-11-10

**Authors:** Montserrat A. Corbera-Hincapie, Kristen S. Kurland, Mark R. Hincapie, Anthony Fabio, Daniel J. Weiner, Sandra C. Kim, Traci M. Kazmerski

**Affiliations:** 1 Department of Pediatrics, University of Pittsburgh School of Medicine, Pittsburgh, PA 15213, USA; hincapiemr@upmc.edu (M.R.H.); daniel.weiner@chp.edu (D.J.W.); sandra.kim@chp.edu (S.C.K.); traci.kazmerski@chp.edu (T.M.K.); 2 School of Architecture, Heinz College of Information Systems and Public Policy, Carnegie Mellon University, Pittsburgh, PA 15213, USA; kurland@andrew.cmu.edu; 3 Department of Epidemiology, University of Pittsburgh Epidemiology Data Center, Pittsburgh, PA 15260, USA; anthony.fabio@pitt.edu

**Keywords:** cystic fibrosis, food insecurity, food deserts, body mass index

## Abstract

Food insecurity (FI) is defined as “the limited or uncertain access to adequate food.” One root cause of FI is living in a food desert. FI rates among people with cystic fibrosis (CF) are higher than the general United States (US) population. There is limited data on the association between food deserts and CF health outcomes. We conducted a retrospective review of people with CF under 18 years of age at a single pediatric CF center from January to December 2019 using demographic information and CF health parameters. Using a Geographic Information System, we conducted a spatial overlay analysis at the census tract level using the 2015 Food Access Research Atlas to assess the association between food deserts and CF health outcomes. We used multivariate logistic regression analysis and adjusted for clinical covariates and demographic covariates, using the Child Opportunity Index (COI) to calculate odds ratios (OR) with confidence intervals (CI) for each health outcome. People with CF living in food deserts and the surrounding regions had lower body mass index/weight-for-length (OR 3.18, 95% CI: 1.01, 9.40, *p* ≤ 0.05 (food desert); OR 4.41, 95% CI: 1.60, 12.14, *p* ≤ 0.05 (600 ft buffer zone); OR 2.83, 95% CI: 1.18, 6.76, *p* ≤ 0.05 (1200 ft buffer zone)). Food deserts and their surrounding regions impact pediatric CF outcomes independent of COI. Providers should routinely screen for FI and proximity to food deserts. Interventions are essential to increase access to healthy and affordable food.

## 1. Introduction

Food insecurity (FI) is an important barrier that has the potential to significantly impact the health and well-being of children and families. The US Department of Agriculture (USDA) defines FI as a household in which there is “limited or uncertain access to adequate food” [1,2]. Some root causes of FI include poverty and food deserts, or low-income census tracts with a substantial number of residents with poor access to retail outlets selling healthy and affordable foods [3]. The USDA further defines low income and low access within a census tract as “a poverty rate of 20 percent or greater, or a median family income at or below 80 percent of the statewide or metropolitan area median family income,” and “at least 500 persons and/or at least 33 percent of the population lives more than 1 mile from a supermarket or large grocery store (10 miles, in the case of rural census tracts),” respectively [4].

Evidence suggests that FI rates among people with cystic fibrosis (CF) may be higher than the general United States (US) population [5]. In CF, optimal growth and nutrition, identified as body mass index (BMI) > 50%, are correlated with better lung function and overall health; therefore, adequate caloric intake is a mainstay of CF care [5,6,7,8]. As people with CF have increased caloric demands (1.5 to 2 times the energy needs of the general population), FI and/or poor access to healthy food options may contribute to inability to achieve and maintain appropriate weight gain and lead to malnutrition in CF [9]. Addressing FI is key because it can independently have detrimental effects on child health, including more frequent hospitalizations, developmental problems, nutritional deficiencies, chronic stress leading to depression/anxiety/toxic stress, and increased long-term mortality resulting from metabolic syndrome, particularly cardiovascular disease [2,10,11].

Although FI screening has increased across many CF centers, there remains limited data on the association between FI, particularly as it relates to food deserts, and CF health outcomes [12]. This study investigated the effects of food deserts and their surrounding regions on health outcomes, including BMI/weight-for-length, percent predicted forced expiratory volume in 1 s (ppFEV1) and hospitalizations secondary to pulmonary exacerbations, in children and adolescents with CF. Additionally, we examined the impact of other neighborhood features that might affect child health by utilizing the Child Opportunity Index (COI).

## 2. Materials and Methods

### 2.1. Setting and Study Design

We conducted a retrospective review of people with CF under 18 years of age at a tertiary level Children’s Hospital center from January 2019 to December 2019. We collected patient demographic information, including age, sex, race/ethnicity, and home address, along with CF health parameters, including year-best BMI percentile or weight-for-length percentile, year-best ppFEV1 for children 6 years and older, CF modulator use, and number of hospitalizations due to pulmonary exacerbation from the US Cystic Fibrosis Foundation Patient Registry (CFFPR).

We geocoded the most recent participant home addresses using a Geographic Information System (GIS), a computerized system that can capture, store, analyze, manage, and present data that are linked to a location. GIS differs from other information systems to address location questions, as it uses multiple layers of geospatial data and advanced spatial statistics, networking, and analysis tools [3].

The Institutional Review Board (IRB) of the University of Pittsburgh approved this study with a waiver of informed consent (protocol number: 20050263).

### 2.2. Area Resources

We used the 2015 Food Access Research Atlas, a USDA database that provides food access data for populations within census tracts and identifies which census tracts are low income and low access, also known as food deserts [13]. It maps food access indicators, such as accessibility to healthy food sources measured by distance or availability of a vehicle, for each individual census tract “using ½-mile and 1-mile demarcations to the nearest supermarket for urban areas, 10-mile and 20-mile demarcations to the nearest supermarket for rural areas” [13]. For the purposes of this study, we used the 1- and 10-mile demarcation for urban and rural areas, which align with the USDA food desert definition. Data from the 2015 Atlas came from the 2010 Decennial Census and the 2014–2018 American Community Survey (ACS).

We used the Child Opportunity Index (COI) 2.0 to examine the impact of neighborhood factors on CF health and to adjust for any confounders in our analysis related to food deserts. COI 2.0 measures neighborhood resources and conditions that play a role in child development and is specific to children. It includes 29 indicators that are sorted into three domains: (a) education; (b) health and environment; and (c) social and economic. Examples of the indicators include, but are not limited to, early childhood education, green space, social and economic resources, employment rate, poverty rate, median household income, and public assistance rate. The COI 2.0 provides an overall opportunity score for each census tract using 2015 US Census Bureau and the American Community Survey data with the lower scores being suggestive of less opportunity. Each domain is also assigned an opportunity score for each census tract. The census tracts are then grouped into high, very high, moderate, low, very low opportunity scores for each domain and overall category. For the purposes of this study, we grouped the COI scores into high/very high versus moderate/low/very low [14].

**Figure 1:**
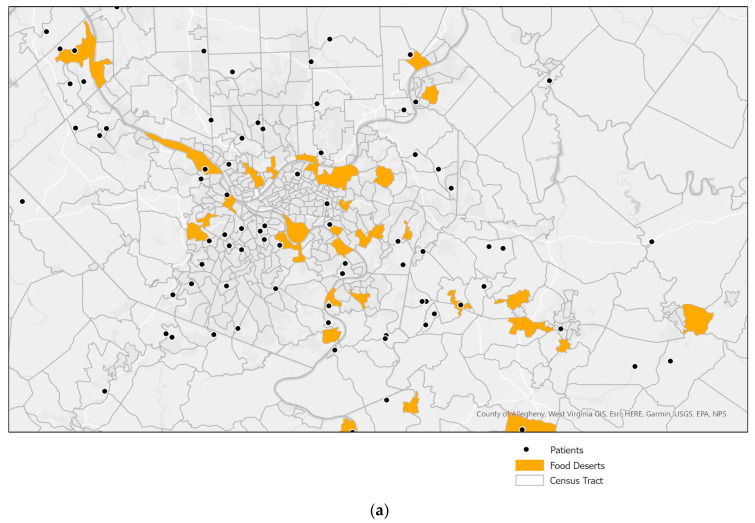

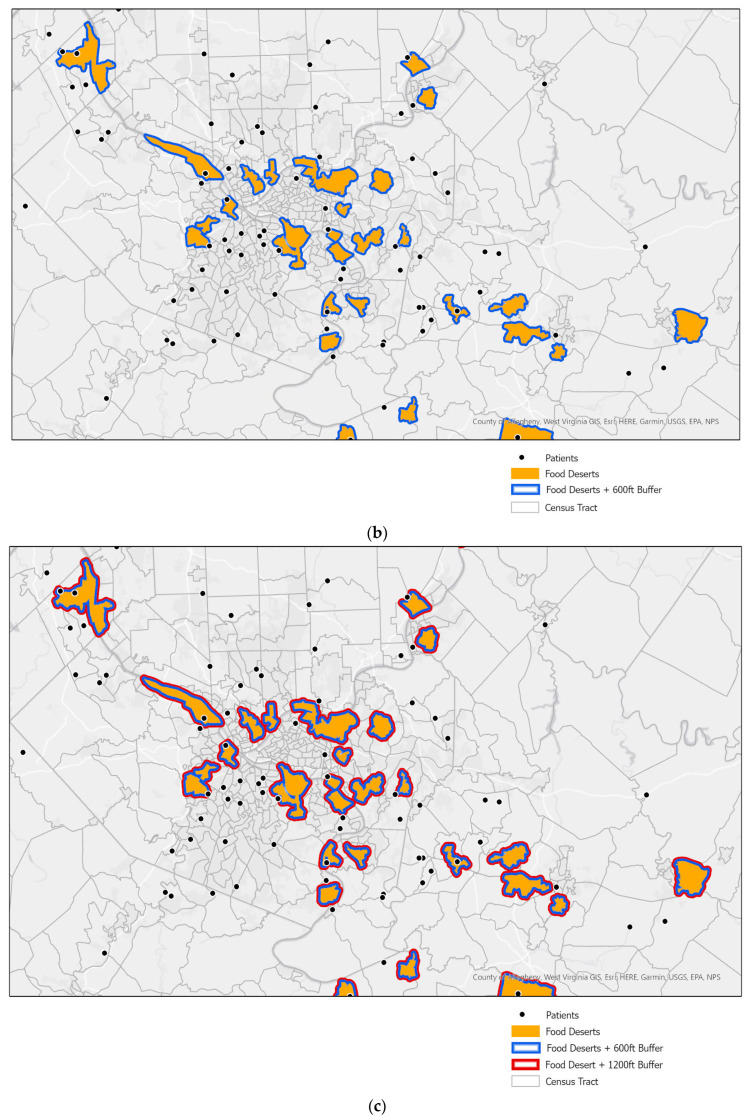
(**a**) Zoomed in spatial overlay of food deserts (Allegheny County, PA, USA) and patient sample. (**b**) Zoomed in spatial overlay of food deserts (Allegheny County, PA, USA) plus a 600 ft buffer and patient sample. (**c**) Zoomed in spatial overlay of food deserts (Allegheny County, PA, USA) plus a 1200 ft buffer and patient sample.

Using GIS software (ArcGIS Pro, Esri, Inc., Redlands, CA, USA), we conducted a spatial overlay analysis using the 2015 Food Access Research Atlas (Figure 1a). We also included buffer zones with a 600-foot (600 ft) and 1200-foot (1200 ft) walkability distance from food deserts to assess if living near a food desert (in addition to living within a food desert) contributed to poor health outcomes (Figure 1b,c). By using a reverse spatial join in ArcGIS Pro, we applied the food desert layer and COI score layer onto the geocoded patient addresses and their health parameters.

### 2.3. Outcome Variables

Our primary health outcome was year-best BMI percentile (for patients above 2 years of age) or weight-for-length percentile (for patients under 2 years of age). Secondary outcomes were lung function (as measured by year-best ppFEV1 for patients that were 6 years and older, and who could adequately and reproducibly participate in the testing) and number of hospitalizations secondary to pulmonary exacerbations. We defined ideal BMI as >50th percentile, ideal ppFEV1 as >90th percentile and significant pulmonary exacerbations if requiring 2 or more hospitalizations in one year. The Cystic Fibrosis Foundation recommends that children and adolescents maintain a BMI or weight for length at or above the 50th percentile, as weight above this threshold has been associated with better ppFEV1 [15]. The CF pulmonary guidelines categorize severity of lung disease with normal FEV1 being greater than 90% predicted [16].

### 2.4. Statistical Analysis

We used GIS software (ArcGIS Pro, Esri, Inc., Redlands, CA, USA) for the geographic data analysis and IBM SPSS Statistics software (Version 28.0, Armonk, NY, USA) for the statistical analysis. We estimated odds ratios and 95% confidence intervals (CI) for associations between food deserts, buffer zones, COI scores, and the three health outcomes (BMI/weight-for-length, ppFEV1, and pulmonary exacerbations requiring hospitalization) using logistic regression.

We created multivariate logistic regression models for each health outcome to assess the individual effects of food deserts (and their buffer zones) and COI scores and both combined. BMI/weight-for-length, ppFEV1, and hospitalizations secondary to pulmonary exacerbations were dependent variables, and food deserts and COI scores were independent variables in the models. Model 1 examined food deserts individually. Model 2 examined COI scores individually and their potential effects on health outcomes. Model 3 examined food deserts adjusting for COI scores. We adjusted each model for covariates, including age, sex (female/male), race/ethnicity (non-Hispanic White/other), and modulator use (yes/no). We repeated this methodology for food deserts plus 600 ft and 1200 ft buffers. We used cross tabulation to observe differences between the several variables that were applied. We defined statistical significance as *p* ≤ 0.05.

## 3. Results

The study included 206 children and adolescents with CF residing in Pennsylvania, West Virginia, and Ohio. Table 1 presents the demographic and clinical characteristics of the patient sample. The average age of the patient sample was 9.5 +/- 5.6 years. About half of the patient population was female (47%). Most of the participants in the sample were White (92.7%), which is consistent with the national US CF population [17].

### 3.1. Model 1: The Individual Impact of Food Deserts and Surrounding Regions on CF Health Outcomes

When accounting for food deserts alone, children and adolescents with CF living in a food desert had 2.9 times the odds (95% CI: 1.1, 8.21, *p* ≤ 0.05) of having a non-ideal BMI as those not living in a food desert. There were no significant increased odds of a non-ideal ppFEV1 or increased hospitalizations from pulmonary exacerbations in food deserts (Table 2).

Children and adolescents with CF living in a food desert or within a 600 ft walkability distance from a food desert had four times the odds (95% CI: 1.54, 10.69, *p* ≤ 0.05) of having a non-ideal BMI as those not living in or 600 ft from a food desert. Additionally, those living in a food desert or within a 600 ft walkability distance from a food desert had three times the odds (95% CI: 0.99, 9.66, *p* ≤ 0.05) to have a non-ideal FEV1 as those not living in or 600 ft from a food desert. There were no significant increased odds of increased hospitalizations from pulmonary exacerbations in food deserts or within a 600 ft walkability distance (Table 2).

Children and adolescents with CF living in a food desert or within a 1200 ft walkability distance from a food desert had 2.7 times the odds (95% CI: 1.16, 6.07, *p* ≤ 0.05) of having a non-ideal BMI as those not living in or 1200 ft from a food desert. There were no significant increased odds of a non-ideal FEV1 or increased hospitalizations from pulmonary exacerbations in food deserts or within a 1200 ft walkability distance (Table 2).

### 3.2. Model 2: The Individual Impact of Childhood Opportunity (COI Scores) on CF Health Outcomes

When accounting for COI scores individually, there were no significant increased odds of a non-ideal BMI, non-ideal FEV1, or increased hospitalization from pulmonary exacerbations in children and adolescents with CF that lived in census tracts that had a moderate/low/very low opportunity score versus a high/very high opportunity score (Table 2).

### 3.3. Model 3: The Impact of Food Deserts and Surrounding Regions on CF Health Outcomes, Adjusting for COI Scores

Children and adolescents with CF living in a food desert had 3.18 times the odds (95% CI: 1.01, 9.4, *p* ≤ 0.05) of having a non-ideal BMI as those not living in a food desert when adjusting for the overall COI score. When adjusting for each individual COI domain, children and adolescents with CF living in a food desert continued to have increased odds of having a non-ideal BMI as those not living in a food desert. There were no significant increased odds of a non-ideal FEV1 or increased hospitalization from pulmonary exacerbations in food deserts when accounting for the overall COI score or any of the three domains (Table 2).

Children and adolescents with CF living in a food desert or within a 600 ft walkability distance from a food desert had 4.4 times the odds (95% CI: 1.6, 12.14, *p* ≤ 0.05) of having a non-ideal BMI and 3.3 times the odds (95% CI: 1.03, 10.84, *p* ≤ 0.05) of having a non-ideal FEV1 as those not living in or 600 ft from a food desert when adjusting for overall COI scores. When adjusting for each individual domain, children and adolescents with CF living in a food desert or within a 600 ft walkability distance continued to have increased odds of having a non-ideal BMI and a non-ideal FEV1 as those not living in or 600 ft from a food desert. There were no significant increased odds of increased hospitalizations from pulmonary exacerbations in food deserts or within a 600 ft walkability distance when accounting for the overall COI score or any of the three domains (Table 2).

Children and adolescents with CF living in a food desert or within a 1200 ft walkability distance from a food desert had 2.8 times the odds (95% Cl: 1.18, 6.76, *p* ≤ 0.05) of having a non-ideal BMI as those not living in or 1200 ft from a food desert when adjusting for overall COI scores. When adjusting for each individual domain, children and adolescents with CF living in a food desert or within a 1200 ft walkability distance continued to have increased odds of having a non-ideal BMI as those not living in or 1200 ft from a food desert. There were no significant increased odds of a non-ideal FEV1 or increased hospitalization from pulmonary exacerbations in food deserts or within a 1200 ft walkability distance when accounting for the overall COI score or any of the three domains (Table 2).

## 4. Discussion

Children and adolescents with CF living in a food desert or within a 600 ft or 1200 ft walkability distance from a food desert have increased likelihood of having a non-ideal BMI/weight-for-length. Patients living within a 600 ft walkability distance from a food desert also had increased odds of having suboptimal lung function. These findings are independent of other childhood opportunity factor or social determinants of health, including healthcare access, education quality, employment, poverty rate, and neighborhood environment, suggesting that the unfavorable health outcomes are driven by food access rather than by alternate social, environmental or health indicators.

Prior studies have demonstrated that the social, economic, and neighborhood environment play a role in the health [18]. Structural factors play a significant role in health inequities and furthermore suggest that “environment restricts freedom of choice, or that behavior is chosen to compensate for unfavorable circumstances” [19]. Poverty is a barrier to healthy foods, a safe environment, and better employment, all of which are predictors of better health [20,21]. Children are not immune from these effects. Children with limited access to healthy foods develop poorer eating habits, placing them at increased risk for developing chronic disease, such as mental health ailments, asthma, and diabetes. Additionally, children, who live in a food insecure household, have increased odds of having fair/poor health reported and of being hospitalized since birth [22].

Our findings are consistent with current literature suggesting that living in a food desert has a negative impact on health [23,24]. It has also been noted that FI contributes to greater healthcare costs, particularly when looking at patients with chronic disease [25]. A systematic review of food deserts found that in addition to limited access to supermarkets, small independent stores and convenience stores charge higher prices for food, compounding the effects of food deserts. Individuals, including children, living in food deserts are at increased risk of developing obesity, in part due to lower fruit and vegetable access [18,21,26,27,28,29]. Some characteristics of neighborhoods that are affected by poor access to grocery stores and healthy foods include lower-income and minority neighborhoods [28]. A cross-sectional study comparing lower income versus higher income neighborhoods found that certain neighborhood characteristics may predispose children to obesity regardless of other demographic or socioeconomic factors [30].

There have been a few studies examining the impact of social determinants of health on health outcomes in people with CF; however, no prior study has looked at the impact of food deserts and their surrounding regions on CF health outcomes. A recent study by Oates and colleagues linked respiratory health in people with CF to state- and area-level characteristics, particularly an association with area resource deprivation and overall state child health [31]. Another study looking at the associations of socioeconomic status with CF health outcomes found that medically indigent individuals with CF suffer more adverse outcomes than the general CF population, including higher mortality and worse pulmonary function and growth [32].

While similar GIS techniques have been used in other chronic disease population studies, this study is one of the first to apply GIS methodology in CF [21,31,33,34]. Importantly, a strength of this study was our use of full home addresses, leading to more precise estimates of geographic linkages to health outcomes. Most studies, including the ones referenced above, use participants’ postal ZIP codes as opposed to full addresses. ZIP codes are helpful in assessing food desert status or level of geography for indices; however, they can span across multiple census tracts consisting of potentially demographically diverse areas and may be less precise due to misclassification of socioeconomic status [35].

Given these findings, it is important to address how we can increase food access for people with CF living in or near food deserts, in addition to identifying individuals at risk. Screening for FI is one potential option; however, some people with CF and families might not answer truthfully due to the stigma associated with FI. Another option is to geographically assess proximity to food deserts as a marker for food resources that might be needed. Expanding the availability of nutritious and affordable food is also key, as limited access to affordable food choices can lead to increased FI. Food deserts need assistance in creating tax incentives, developing and equipping grocery stores, small retailers, corner markets, and farmer’s markets, along with partnering with community allies that can enhance the ability to connect families to resources [36]. Driving economic growth in food deserts could lead to indirect health benefits. One example of a proposed intervention is the Healthy Food Financing Initiative implemented by the US government, which utilizes funding by the federal government to bring stores and food retailers to underserved communities [37]. Additionally, similar to farmer’s markets, mobile food markets expand access to larger areas, but can cover more area in a shorter amount of time [37,38,39]. The Green Grocer mobile farmer’s market, piloted by the Greater Pittsburgh Community Food Bank, is an example of a program that has the potential to reduce geographically based disparities in food deserts. It is owned and operated by a food bank and designed to be affordable and accept multiple forms of payment, including Electronic Benefits Transfer cards that have Supplemental Nutrition Assistance Program (SNAP) benefits [38,39]. Another successful program is the Just Harvest organizations, which targets FI at the individual, community, and national level by partnering with community organizations to develop grocery stores in food deserts. Additionally, schools and child nutrition programs are profoundly important in providing food access to children. Some of these programs include the School Breakfast Program, National School Lunch Program, and Afterschool Nutrition Program, all of which have been shown to have a positive effect on the health of food insecure children [40]. Social workers play an invaluable role in connecting patients and their families to local and federal food programs, such as the ones mentioned above. This is especially true for patients with chronic diseases, such as CF, because they are seeing the specialist on a more frequent basis, creating more opportunities for FI screening, resource distribution, and follow up. It is also important to acknowledge that technology can play a vital role in decreasing barriers to food access, as online grocery sales have become increasingly more widely available. Delivery services can be of great benefit for patients and families, who do not have a vehicle and are reliant on public transportation or ride sharing. However, this technology can be hindered by delivery and service fees, making this service inaccessible to an individual already facing food insecurity [41]. The Baltimore Ride Share Project is a program that was piloted in Baltimore in 2019, which provides transportation services to and from supermarkets at a discounted rate via a ride share service for residents living in food deserts. This is a program that can be replicated in other cities and decrease barriers to food access for individuals living in food deserts [42]. The CF community and advocacy organizations should consider partnering with similar organizations that are near CF care centers where a high proportion of their patients are either food insecure and/or live in or near a food desert. Additionally, government agencies can adopt the Healthy Food Priority Area (HFPA) designation, an initiative developed by Baltimore in 2018 that prioritizes certain areas for action against food insecurity. Each census tract is assigned a score for food availability, food access, and food utilization, and those with higher scores are designated HFPAs. This initiative helps identify high-risk communities and prioritize a city’s action against food insecurity [41].

This study has several limitations. First, it examines a small sample from a single center in the Mid-Atlantic/Northeast US and, thus, may not be generalizable to other regions. Additionally, it does not capture everyone who experiences FI, as there are people with CF who live outside of food deserts who are food insecure. Future work may consider utilizing similar methods to analyze the health impacts of food deserts on people with CF nationally. Moreover, exploring the perspectives of people with CF and their families who experience FI and/or reside in food deserts may help target future interventions to increase access to healthy and affordable food.

## 5. Conclusions

Food deserts and their surrounding regions impact pediatric CF outcomes, particularly BMI. CF teams should routinely screen for FI and proximity to food deserts, and development of novel interventions are essential to increase access to healthy and affordable food. The methods used in this study can serve as a model to assess the association between food deserts and health outcomes more broadly in CF and in other chronic gastrointestinal diseases. Furthermore, as prior research has shown that FI may be strongly predictive of chronic illnesses, these results may have widespread applicability, as nutrition is one of the mainstays of therapy for many chronic diseases [43].

## Figures and Tables

**Table 1:** Characteristics of the CF patient sample.

Characteristics	Overall	Food Desert	Food Desert + 600 ft Buffer	Food Desert + 1200 ft Buffer
Total	206	17	20	29
**Demographics**				
Age (range 0–18 years)	9.5 * (5.6)	8.7 * (5.5)	9.5 * (5.5)	9.8 * (4.9)
Female, %	47%	47%	45%	52%
White, %	92.7%	94%	95%	93%
**Modulator Use**, %	11.6%	18%	20%	14%
**Clinical Outcomes**				
BMI/weight-for-length, percentile	63 * (24.4)	56.6 * (29.7)	53.5 * (28.5)	56.8 * (28.0)
ppFEV1 (>6 years old)	95.9 * (17.1) n = 141	92.5 * (17.2) n = 11	87.2 * (18.8) n = 14	90 * (16.9) n = 22
Pulmonary Exacerbations	0.5 * (1.2) Range: 0–8	0.7 * (2.2) Range: 0–6	0.8 * (1.4) Range: 0–6	0.6 * (1.2) Range: 0–6

* Mean (standard deviation).

**Table 2:** Multiple Regression Models of CF Outcomes: Food Deserts, Surrounding Regions, and COI.

Health Outcome	COI	Food Desert	Food Desert +	Food Desert +
			600 ft Buffer	1200 ft Buffer
**BMI**				
Model 1 ^a^	1.07 (0.59, 1.94) *p*-value 0.832	2.9 (1.1, 8.21) *	4.06 (1.54, 10.69) *	2.65 (1.16, 6.07) *
OR (95% CI)		*p*-value 0.039	*p*-value 0.005	*p*-value 0.021
Model 2 ^b^				
OR (95% CI)				
Model 3 ^c^		3.18 (1.01, 9.40) *	4.41 (1.60, 12.14) *	2.83 (1.18, 6.76) *
OR (95% CI)		*p*-value 0.036	*p*-value 0.004	*p*-value 0.020
**ppFEV1 (6 years and older)**				
Model 1 ^a^	0.99 (0.49, 2.02) *p*-value 0.984	1.79 (0.51, 6.32)	3.09 (0.99, 9.66) *	1.90 (0.74, 4.92)
OR (95% CI)		*p*-value 0.363	*p*-value 0.050	*p*-value 0.184
Model 2 ^b^				
OR (95% CI)				
Model 3 ^c^		1.90 (0.51, 7.14)	3.33 (1.03, 10.84) *	2.02 (0.75, 5.46)
OR (95% CI)		*p*-value 0.340	*p*-value 0.045	*p*-value 0.166
**Hospitalizations for** **Pulmonary Exacerbations**				
Model 1 ^a^	1.01 (0.41, 2.53) *p*-value 0.976	2.22 (0.53, 9.25)	2.49 (0.69, 8.95)	1.19 (0.35, 4.05)
OR (95% CI)		*p*-value 0.273	*p*-value 0.163	*p*-value 0.778
Model 2 ^b^				
OR (95% CI)				
Model 3 ^c^		2.42 (0.52, 11.15)	2.63 (0.69, 10.01)	1.20 (0.34, 4.31)
OR (95% CI)		*p*-value 0.258	*p*-value 0.156	*p*-value 0.777

^a^ Model 1: Food Desert +/- Buffer Zone Alone; ^b^ Model 2: COI Alone; ^c^ Model 3: Food Desert +/- Buffer Zone adjusted for COI Overall Score; OR: odds ratio; CI: confidence interval; * Significant estimate at *p* ≤ 0.05.

## Data Availability

The data presented in this study are available on request from the corresponding author.
